# Identification of Novel Knockout Targets for Improving Terpenoids Biosynthesis in *Saccharomyces cerevisiae*

**DOI:** 10.1371/journal.pone.0112615

**Published:** 2014-11-11

**Authors:** Zhiqiang Sun, Hailin Meng, Jing Li, Jianfeng Wang, Qian Li, Yong Wang, Yansheng Zhang

**Affiliations:** 1 CAS Key Laboratory of Plant Germplasm Enhancement and Specialty Agriculture, Wuhan Botanical Garden, Chinese Academy of Sciences, Wuhan, China; 2 Institute of Plant Physiology and Ecology, Shanghai Institutes for Biological Sciences, Chinese Academy of Sciences, Shanghai, China; 3 GIAT-HKU joint Center for Synthetic Biology Engineering Research, Guangzhou Institute of Advanced Technology, Chinese Academy of Sciences, Guangzhou, China; CNR, Italy

## Abstract

Many terpenoids have important pharmacological activity and commercial value; however, application of these terpenoids is often limited by problems associated with the production of sufficient amounts of these molecules. The use of *Saccharomyces cerevisiae* (*S. cerevisiae*) for the production of heterologous terpenoids has achieved some success. The objective of this study was to identify *S. cerevisiae* knockout targets for improving the synthesis of heterologous terpeniods. On the basis of computational analysis of the *S. cerevisiae* metabolic network, we identified the knockout sites with the potential to promote terpenoid production and the corresponding single mutant was constructed by molecular manipulations. The growth rates of these strains were measured and the results indicated that the gene deletion had no adverse effects. Using the expression of amorphadiene biosynthesis as a testing model, the gene deletion was assessed for its effect on the production of exogenous terpenoids. The results showed that the dysfunction of most genes led to increased production of amorphadiene. The yield of amorphadiene produced by most single mutants was 8–10-fold greater compared to the wild type, indicating that the knockout sites can be engineered to promote the synthesis of exogenous terpenoids.

## Introduction

Terpenoids, which are candidate drugs and fragrances, are important secondary metabolites derived from the universal precursors isopentenyl diphosphate (IPP) and its isomer dimethylallyl diphosphate (DMAPP) [1], [2]. The low concentrations present in host organisms and the complicated structure of many terpenoids limits their suitability for commercial applications [3]. Although plant cell cultures and transgenic plants have been made to improve isoprenoid biosynthesis [4], [5], plant-based efforts are less likely to supply sufficient commercial-scale quantities of terpenoids. The development of biological synthesis presented a promising alternative for producing large quantities of plant terpenoids in microorganisms such as *Escherichia coli* and *Saccharomyces cerevisiae* [6], [7]. Compared to bacteria, *S. cerevisiae* is more advantageous for synthesizing plant terpenoids owing to its ability to express plant cytochrome P450 enzymes [8], [9]. Monoterpenes, sesquiterpenes and diterpenes have been engineered using yeast cells as a living factory [10]–[12]. Very recently, the anti-malarial drug artemisinin intermediate artemisinic acid was produced in *S. cerevisiae* at a titer of 25 g·L^−1^, which encourages the use of industrial manufacture of plant terpenes in microbial systems [13]. A sufficient supply of endogenous IPP for the heterologous terpenoid pathway is a crucial issue for metabolic engineering of plant terpenes in yeast cells [7], [14]. Traditional engineering strategies designed to increase carbon flux toward IPP biosynthesis have relied heavily on amplifying the transcripts of several genes in the upstream pathway to IPP, often resulting in an unbalanced carbon flux and accumulation of toxic intermediates that inhibit yeast growth [10], [15]. For this reason, there are two important challenges to be met by traditional engineering strategies. First, the simple up or down regulation of genes on the isoprenoid pathway might cause suboptimal yeast viability [16]. Second, owing to complex interactions between intracellular fluxes, modulation of gene targets solely on the isoprenoid pathway might be less able to direct carbon fluxes into the production of desired molecules. To overcome these difficulties, a genome-scale metabolic model of *S. cerevisiae*, iMM904, has been developed and used for metabolic engineering in yeast cells [17], [18]. The aim of this study was to discover novel gene knockout targets outside the isoprenoid pathway that could improve isoprenoid fluxes in *S. cerevisiae* while not inhibiting yeast growth. First, *in silico* strategies based on iMM904 were used to identify possible knockout targets. To test their suitability, experiments were designed to knock out these targets from an *S. cerevisiae* strain maintained in our laboratory, which was then engineered to produce the sesquiterpene amorphadiene. Production of amorphadiene was measured to investigate the impact of these gene deletions on isoprenoid pathway fluxes. We described ten novel gene knockout targets located in *S. cerevisiae* central metabolism pathways and each knockout caused an 8–10-fold increase of the levels of downstream heterologous terpenoids.

## Materials and Methods

### Materials

Microbial growth medium was purchased from OXOID LTD (Basingstoke, UK). Enzymes and kits were purchased from Fermentas International (Burlington, Canada), and Takara Biotechnology Co., Ltd. (Takara Dalian, China). Caryophyllene and *n*-pentadecane standards were purchased from TCI (Shang Hai, China) and dodecane was purchased from Sigma-Aldrich (St Louis, MO).

### In silico prediction of knockout sites

The computational platform was constructed according to Schellenberger et al. [19] and Becker et al. [20]. All simulations and calculations were based on iMM904, the genome-scale metabolic model of *S. cerevisiae* [18]. Flux balance analysis (FBA) and minimization of metabolic adjustment (MOMA) methods were used for the prediction of knockout targets. The details regarding the calculation were described in the supplemental data.

### Construction of deletion cassettes and yeast expression vectors

Single mutants were obtained by one-step integration of deletion cassettes into yeast genome. Each deletion cassette consisted of two homologous regions of the corresponding target gene and one selectable marker flanked by two loxp sites. The selectable marker section was composed of the TEF gene promoter, the antibiotic marker gene KanMX, and the TEF terminator. In the deletion cassette, the selectable marker section was cloned between two homologous regions of the target gene. The *CRE* (recombinase gene) expression vector pYPT2 was constructed to remove the selectable marker integrated into the yeast genome during the preparation of the knockout mutants. The recombinase gene *CRE* complete sequence was PCR-amplified from the vector pSH47 [21] and introduced into the vector p416 GPD (ATCC no. 87360) under *SalI* and *BamHI* sites to give the construct pYPT2. The cDNA coding amorphadiene synthase (ADS) was PCR-amplified from the *Artemisia annua* leaves and cloned into the vector pESC-HIS (Stratagene) to obtain the construct pYPT4. The primers used for the PCRs were listed in Table S1 in File S1.

### Yeast strain construction and cultivation

The parental yeast strain WAT11 [22] was used in this study for constructing mutants. Yeast single mutants were obtained by one-step integration of deletion cassettes [23], which were transformed into WAT11 cells made competent by lithium acetate [24] and integrated into the genome. The selectable marker in the deletion cassettes was removed by transforming a recombinase expression vector pYPT2 into the transformants, and the recombinase acted on loxp sites to cut off the selectable marker [25]. Finally, single mutants without a selectable marker were obtained. The method used to construct double mutants was the same as described above for the construction of single mutants. One single mutant was used as the parental strain to transform another deletion cassette following the procedures used for the construction of the single mutants. All the mutant strains made in this study were given in Table 1.

**Table 1:** Yeast strains used and constructed in this study.

Strains name	Genotype
WAT11	MATα (*leu2-3,112 trp1-1 can1-100 ura3-1 ade2-1 his3-11,15*)
YS1	WAT11*alt2*Δ
YS2	WAT11*ctp1*Δ
YS3	WAT11*gre3*Δ
YS4	WAT11*hxk1*Δ
YS5	WAT11*hxk2*Δ
YS6	WAT11*idp1*Δ
YS7	WAT11*ser1*Δ
YS8	WAT11*ser2*Δ
YS9	WAT11*ser3*Δ
YS10	WAT11*ser33*Δ
YS11	WAT11*sor1*Δ
YD1	WAT11*hxk2*Δ*ser3*Δ
YWTA	WAT11::*his3*::*ads*
YSA1	WAT11*alt2*Δ::*his3::ads*
YSA2	WAT11*ctp1*Δ::*his3::ads*
YSA3	WAT11*gre3*Δ::*his3::ads*
YSA4	WAT11*hxk1*Δ::*his3::ads*
YSA5	WAT11*hxk2*Δ:*:his3::ads*
YSA6	WAT11*idp1*Δ::*his3::ads*
YSA7	WAT11*ser1*Δ::*his3::ads*
YSA8	WAT11*ser2*Δ:*:his3::ads*
YSA9	WAT11*ser3*Δ::*his3::ads*
YSA10	WAT11*ser33*Δ::*his3::ads*
YSA11	WAT11*sor1*Δ::*his3::ads*
YDA1	WAT11*hxk2*Δ*ser3*Δ::*his3::ads*

Yeast strains were cultured at 30°C with rotation at 250 rpm. For screening mutants, the yeast transformants were grown in YPD medium consisting of YPD medium supplemented with 200 mg·L^−1^ G418. Strains harboring expression vectors were grown in SD medium containing 0.67% (w/v) yeast nitrogen base, 2% glucose unless indicated otherwise and amino acids without uracil or without histidine. For inspecting yeast growth property, the single colony was inoculated into 5 ml of YPD medium and grown overnight. Next day, the cultures were transferred into 20 ml of fresh YPD medium in 50 ml flasks and the initial *A*_600_ was regulated to 0.06. All cultures were sampled at the scheduled time points and *A*_600_ was measured with a spectrophotometer.

For the production of amorphadiene, an overnight pre-culture grown in SD medium without histidine was inoculated into 25 ml of induction medium supplemented with galactose as sole carbon source at an *A*_600_ value of 0.8. A two-phase culture was used and the organic phase (dodecane) was 16% of the total volume [26]). Strains with the ADS expression vector were grown in induction medium and amorphadiene was enriched synchronously by the organic phase to prevent loss of volatile amorphadiene. The pattern of amorphadiene accumulation was investigated to determine the optimum sampling time point. Five time points were chosen (12, 24, 48, 72 and 96 h-post induction) and samples of the organic phase were taken at each time point for GC-MS analysis. Once the appropriate sampling time point was set, the production of amorphadiene among all the strains was then compared at that time point.

### GC-MS measurement of amorphadiene

Quantification of amorphadiene was done by GC-MS. The instrument was an Agilent 7890A/5975C equipped with an HP-5 capillary column (30 m×0.25 µm). The injection port temperature was 250°C and the column temperature was 280°C. The carrier gas was helium at a flow rate of 1 ml·min^−1^. The organic phase samples (200 µl) were analyzed using the following temperature program: 100–200°C at 5°C·min^−1^; 200–250°C at 25°C·min^−1^ [26]. Amorphadiene was identified by analyzing the mass spectra. *n*-Pentadecane (0.05%) was added as an internal standard to quantify the concentration of amorphadiene. Caryophyllene was used as an equivalent standard of amorphadiene for the quantification [6], [27] because amorphadiene standard is not commercially available.

## Results

### Knockout targets with the potential of improving the IPP supply were predicted

In the previous work, we found that the maximum theoretical yield of IPP produced by *S. cerevisiae* via MVA pathway is 0.695 mol IPP per mol glucose [28]. That is, up to 69.5% of the total carbon flux of the metabolic network is diverted into the MVA pathway for IPP formation. The results were obtained under setting IPP biosynthesis rate as the objective. When growth rate was set as the objective, the carbon flux towards IPP synthesis was zero. The wild type strain, however, takes growth, not IPP synthesis, as the native objective; therefore, strategies are needed to redesign the nodes of the metabolic network to redirect the cellular fluxes to achieve a greater yield of IPP and Flux Distribution Comparison Analysis (FDCA) was proposed for this purpose. Based on the iMM904 model, FDCA was used to find the potential key nodes (reactions) for improving IPP supply by setting the maximum biomass formation as one objective and the maximum IPP synthesis rate as the other. The flux distributions of the metabolic network with maximum growth rate or maximum IPP synthesis rate as the objective and the difference between them are shown in Figure S1 in File S1. For the maximum growth rate as an objective, carbon flow was diverted from central metabolism to generate biomass precursors (amino acids, nucleotides, etc.) and energy, resulting in high fluxes of pentose phosphate pathway and citric acid cycle but zero of IPP formation. For the maximum IPP synthesis rate as an objective, however, the majority of carbon atoms flowed into the MVA/DXP pathway and generates IPP, while a small part was diverted for redox balance, resulting in a totally inactive mode of TCA cycle (*S. cerevisiae*).

**Figure 1:**
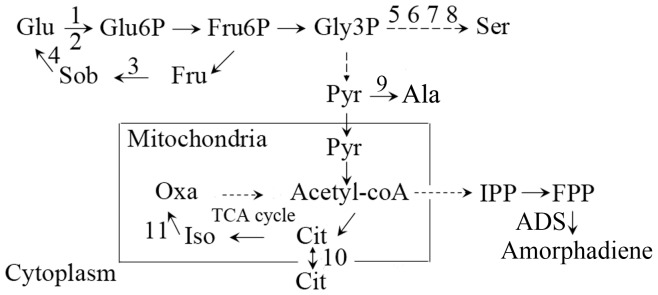
The candidate genes are located mainly in the Embden–Meyerhof–Parnas (EMP) pathway and the TCA cycle, and are associated with amino acid metabolism, and fructose and mannose metabolism. Numerals refer to genes to be disrupted in this work: 1, hexokinase I; 2, hexokinase II; 3, sorbitol reductase; 4, sorbitol de- hydrogenase; 5, phosphoglycerate dehydrogenase; 6, phosphoglycerate dehydrogenase; 7, 3-phosphoserine aminotransferase; 8, 3-phospho- serine phosphatase; 9, L-alanine transaminase; 10, mitochondrial citrate transport; 11, isocitrate dehydrogenase. Abbreviations represent: Glu, glucose; Glu6P, glucose-6p; Fru6P, fructose-6p; Fru, fructose; Sob, sobitol; Gly3p, glyceraldeyde-3p; Ser, serine; Ala, alanine; Pyr, pyruvate; AceCoA, acetyl-CoA; Cit, citrate; Iso, isocitrate; Oxa, oxaloacetate;ADS, amorphadiene synthase.

Node reactions were found by comparing the flux distribution under biomass formation and IPP production conditions. Candidate genes for knockout were tested by minimization of metabolic adjustment (MOMA) method to exclude any lethal target. The potential knockout targets for *S. cerevisiae* (with predicted growth rate more than 99% after knockout) were predicted as shown in Figure 1. All the knockout targets locate in *S. cerevisiae* central metabolism pathways: targets 1–4 code for enzymes in the glucose metabolism pathway; targets 5–9 encode enzymes in amino acid biosynthesis, and targets 10 and 11 locate in the tri-carboxylic acid (TCA) cycle (Figure 1). Our *in silico* analysis predicted that the dysfunction of these sites would not inhibit yeast growth but could increase the supply of IPP, the common precursor for the biosynthesis of downstream terpenoids.

### Single knockout mutant increased the yield of amorphadiene in 8–10 folds compared to the wild type strain

Each of the predicted sites of *S. cerevisiae* was experimentally disrupted. As shown in Figure S2 in File S1, all the single mutants had basically similar growth pattern to the wild type WAT11 strain except that the YS5 mutant grew slightly faster at the later stages and reached the stable phase a little earlier. The results were consistent with the *in silico* prediction above that the gene deletion would not decrease yeast growth.

**Figure 2:**
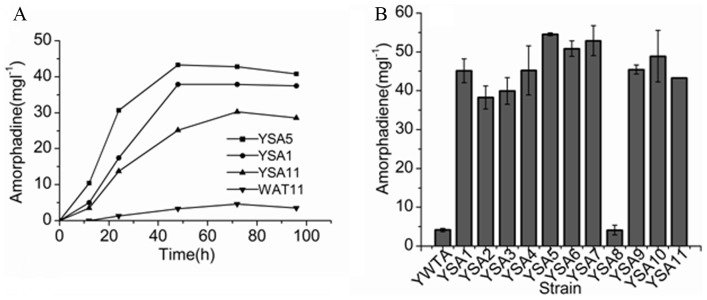
The production of amorphadiene in the wild type WAT11 strain and single mutants. (A) The accumulation pattern of amorphadiene by the wild type WAT11 strain and the three single mutants (YS1,YS5, YS11) at 12, 24, 48, 72 and 96 h after the induction; (B) The production of amorphadiene by each single mutant compared to the wild type WAT11 strain at the 72 h post-induction. The level of amorphadiene at the initiation point was set as zero, and the levels at other time points were normalized thereto.

To examine the effect of the gene deletions on the expression of exogenous terpenoids' biosynthesis, amorphadiene was chosen as the testing terpenoid and amorphadiene synthase was expressed in the mutants and wild type WAT11 strain. To compare the yield of amorphadiene, it is necessary to understand the accumulation pattern of amorphadiene produced by these strains via a time course during the cultivation of yeast cells. Three mutated strains were chosen for this experiment on the basis of their metabolic category. YS1, YS5 and YS11 correspond to alanine metabolism, the glycolysis/gluconeogenesis pathway, and fructose and mannose metabolism, respectively; WAT11 was used as the control. These four strains had similar patterns of amorphadiene accumulation but the maximal level of amorphadiene varied among them. Amorphadiene production by single mutants was much higher compared to the wild type WAT11 strain obviously (Figure 2A). On the basis of the accumulation pattern, setting 72 h as the optimum sampling time was reasonable for comparing the productivity of amorphadiene in all the strains. As shown in Figure 2B, the production of amorphadiene by the single mutants, except YSA8, was about 10-fold greater compared to the wild type WAT11 strain. The highest yield (up to 54.55 mg·L^−1^), which was from YS5, was about 12-fold greater compared to WAT11 (4.14 mg·L^−1^). The amorphadiene yields from YS8 (about 4.13 mg·L^−1^) and WAT11 were essentially identical. The results suggested that most gene deletions increased the yield of amorphadiene, likely because metabolic network flux change in the mutants allowed more carbon to flow into the IPP supply.

### Double mutant did not further increase the production of amorphadiene compared to the single mutant

**Figure 3:**
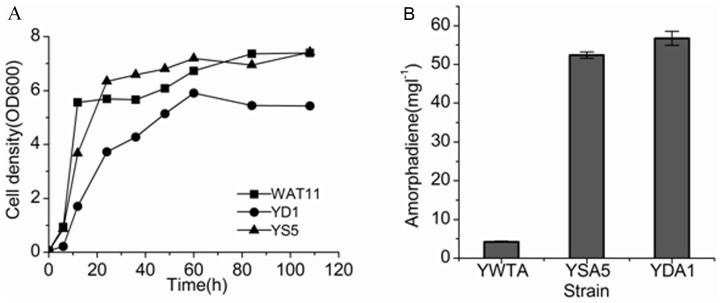
Comparison of yeast cell growth (A) and amorphadiene production (B) between the single mutant YS5, the double mutant YD1 and the wild type strain WAT11.

The high improvement in amorphadiene production by single mutants prompted us to investigate whether double mutants would further increase the level of amorphadiene. To test this hypothesis, the genes *HXK2* and *SER3* were simultaneously disrupted and the resulting double mutant YD1 strain was constructed. The growth property of YD1 was compared to the single mutant YS5 (the deletion of HXK2) and wild type WAT11 strain. As shown in Figure 3A, the growth of the double mutant YD1 strain was depressed markedly in comparison to the single mutant YS5 and WAT11 strains, although the deletion of either HXK2 or SER3 did not decrease the yeast growth (Figure S1 in File S1). For the production of amorphadiene, the double mutant YD1 strain did not increase the level of amorphadiene significantly compared to the single mutant YS5 (Figure 3B). The amorphadiene yield from YD1 was about 11-fold and the yield from YS5 was about 10-fold greater compared to that from the wild type WAT11 strain (Figure 3B). These results suggested that the double mutant did not increase the carbon flux into the IPP supply compared to the single mutant. The construction of multi-mutants by simple combination of single mutants was not expected to be worth pursuing owing to the unexpected imbalance between yield promotion and growth inhibition.

## Discussion

The 25 g·L^−1^ titer of artemisinic acid engineered in *S. cerevisiae* makes it a robust candidate for the production of high-value chemicals on a commercial scale [13]. The objective of this study was to identify key *S. cerevisiae* gene knockout targets that could improve isoprenoid fluxes. With the aid of genome scale-based *in silico* strategies, we identified experimentally, for the first time, ten knockout genes with each causing 8–10-fold improvement of isoprenoid production in *S. cerevisiae*. The gene targets described here are involved in primary metabolism, including glucose metabolism, amino acid (serine and alanine) biosynthesis and the mitochondrial tricarboxylic acid (TCA) cycle. Deletion of these genes might enhance the supply of acetyl-CoA to the MVA pathway in *S. cerevisiae*, which ultimately affects downstream exogenous terpenoid production. Among the positive targets identified, there are five genes encoding enzymes related to oxidative or reduced reactions: SOR1 and Gre3 are NAD^+^ and NADH-specific dehydrogenase/reductase for sorbitol and fructose metabolism, respectively [29], [30]; IDP1 corresponds to the mitochondrial isocitrate dehydrogenase isoenzyme in the TCA cycle and its activity is NADP^+^-dependent [31], [32]; SER3 and SER33 are phosphoglycerate dehydrogenases in the serine biosynthesis pathway [33]. It was not clear why disruption of these redox enzymes stimulated the isoprenoid biosynthesis pathway. However, the synthesis of some primary and secondary metabolites has been reported to be influenced readily by the availability of NAD(P)^+^, NAD(P)H and ATP; so, loss of these enzymes might perturb the redox balance in cells allowing major changes of cellular flux distribution. Deletion of SER33 was reported to improve carotenoid production but had no effect on the biosynthesis of a sesquiterpene, engineered in *S. cerevisiae*, which is in contrast to the results of this study [16]. We showed that deletion of these redox enzymes had no, or a less detrimental, effect on yeast growth on glucose. SOR1 and Gre3 have been described for sorbitol and fructose metabolism and their expression levels are inducible by various stresses [34], [35]. Because glucose or galactose was used as sole carbon source in this study, expression of SOR1 and Gre3 might not be essential and their deletion would not lead to a deleterious phenotype. For the isocitrate dehydrogenases, yeast possesses several isocitrate dehydrogenase isoenzymes (IDP1–3 and IDH) and only IDP1 and IDH are considered to be localized to the mitochondria [31]. Under normal conditions, the contribution of IDH is of major importance, whereas IDP1 does not participate in the TCA-based respiratory process. Therefore, not unexpectedly, the IDP1 knockout strain showed growth similar to the wild type, which is consistent with earlier reports. SER3 and SER33 are enzymes in the glycolytic pathway for the biosynthesis of serine [33]. In yeast, there are two alternate metabolic fluxes toward serine biosynthesis: the phosphorylated pathway from glycolytic intermediates and the glyoxylate pathway from TCA cycle intermediates. The glyoxylate pathway might compensate for the loss of SER3/SER33 to form serine, which made the knockout strains grow normally. In addition, the genes encoding hexokinase (HXK1 and −2), aminotransferase (ALT2 and SER1) and the citrate transporter (CTP1) are positive knockouts for improving isoprenoid fluxes. HXK1 and −2 are isoenzymes acting on glucose metabolism in the upper glycolytic pathway [36], [37]. It was reported that deletion of HXK2 led to a 10-fold increase of HXK1 expression in *S. cerevisiae* strains [38]. Therefore, a possible explanation for the results of this study is deletion of one HXK isoenzyme might trigger a marked increase in the expression of the other, causing reinforcement of the glycolysis flux to the MVA pathway and further experiments are needed to test this. ALT2 and SER1 are associated with biosynthesis of alanine and serine, respectively, whereas CTP1 appears to catalyze the efflux of citrate across the mitochondrial inner membrane in the TCA cycle [39]. The reason why these gene knockouts redirect fluxes to the isoprenoid pathway are not clear. Especially for ALT2, its physiological role *in vivo* is not clear, although it is translated in yeast cells. ALT2 was reported to have no alanine aminotransferase activity, whereas its homologous enzyme ALT1 does *in vivo* [40], [41]. For CTP1, earlier Southern blot experiments indicated only one copy of the CTP1 gene exists in the *S. cerevisiae* genome. However, deletion of the CTP1 gene did not cause any deleterious effect on yeast viability [42], which was observed also in this study. It appears there are other transporters in the TCA cycle compensating for the absence of CTP1 from *S. cerevisiae* [39].

Most targets in this study have isoenzymes and their role *in vivo* is not clear; therefore, it would be difficult to predict knockout targets on the basis of traditional biochemical reactions. The work described here presented an example of the power of *in silico* strategies based on a genome-scale model for application in metabolic engineering. It will be interesting, of course, to predict multiple knockout targets for further improving isoprenoid fluxes using an *in silico* strategy. Owing to the complexity of the interactions between carbon fluxes, the simple combination of the top knockout targets would result in suboptimal performance; in this work, the combination of HXK2 and SER3 failed to obtain a significantly higher concentration of amorphadiene compared to a single knockout.

## Supporting Information

File S1 **This file contains supplemental data including Figure S1, Figure S2, and Table S1.** Figure S1, Flux distribution comparison analysis. (A) The flux distribution of the metabolic network with maximum growth rate as the objective; (B) The flux distribution of the metabolic network with maximum IPP formation as the objective; (C) The difference of flux distribution of the metabolic network between A and B. Figure S2, The growth property of the wild type WAT11 strain and single mutants. Table S1, Primers used in this study.
(DOCX)
