# Supplementary material for: Identification of Novel Knockout Targets for Improving Terpenoids Biosynthesis in Saccharomyces cerevisiae
Source: PLoS One. 2014 Nov 11;9(11):e112615. doi: 10.1371/journal.pone.0112615 (PMC4227703; doi:10.1371/journal.pone.0112615)
Supplement: File S1 — This file contains supplemental data including Figure S1, Figure S2, and Table S1. Figure S1, Flux distribution comparison analysis. (A) The flux distribution of the metabolic network with maximum growth rate as the objective; (B) The flux distribution of the metabolic network with maximum IPP formation as the objective; (C) The difference of flux distribution of the metabolic network between A and B. Figure S2, The growth property of the wild type WAT11 strain and single mutants. Table S1, Primers used in this study. (DOCX) [file pone.0112615.s001.docx]

**Supplemental data**

**Identify novel knockout targets for improving terpenoids biosynthesis in *Saccharomyces cerevisiae***

Zhiqiang Sun ^1&^, Hailin Meng^2&^, Jing Li^1^, Jianfeng Wang^2^, Qian Li ^1^, Yong Wang^2*^, Yansheng Zhang^1*^

^1^ CAS Key Laboratory of Plant Germplasm Enhancement and Specialty Agriculture, Wuhan Botanical Garden, Chinese Academy of Sciences, Wuhan, China

^2^Institute of Plant Physiology & Ecology, Shanghai Institutes for Biological Sciences, Chinese Academy of Sciences, Shanghai, China

^*^Corresponding author. Email address: [yongwang@sibs.ac.cn](mailto:yongwang@sibs.ac.cn), [zhangys@wbgcas.cn](mailto:zhangys@wbgcas.cn)

& These authors contributed equally to this work.

**Flux balance analysis (FBA) and minimization of metabolic adjustment (MOMA)**

FBA method and MOMA method are described in detail elsewhere [[22](#_ENREF_22),[23](#_ENREF_23)]. Defined specifically by iMM904, the upper bound of a reaction is usually set to 1 mol•gDCW^-1^•h^-1^ (gDCW, gram dry cell weight) and the lower bound is usually set to –1.0 mol•gDCW^−1^•h^−1^ of a reversible reaction or zero of an irreversible reaction. Solving the linear programing problem of FBA or the quadratic programming problem of MOMA might derive from the value of the objective function (such as specific growth rate) and the corresponding flux distribution.

**Flux distribution comparison analysis (FDCA)**

The FDCA developed for potential target gene mining for strain improvement was constructed on the basis of FBA. Maximization of biomass formation and targeted product (i.e. IPP) were each selected as the objective function under the same conditions. The corresponding flux distributions were obtained after FBA analysis. These two flux distributions were compared to each other to find metabolic nodes (reactions) with significant difference. Specifically, a new vector was defined as:

*v*_diff_ = *v*_product_ – *v*_biomass_

where *v*_product_ is the flux distribution with target product synthesis rate as the objective function, *v*_biomass_ the flux distribution with biomass formation rate as the objective function, and *v*_diff_ a vector representing difference between these two flux distributions. The significant differences can be detected by the *v*_diff_ analysis to identify the potential reaction (gene) targets for strain improvement.

One circumstance would be considered for the *i*th reaction with significant difference: if *v*_i,biomass_ is a high value while *v*_i,product_ equals to 0, the corresponding reaction enzyme(s) may need to be deleted, or the corresponding gene(s) may be the knockout site(s). Lastly, those lethal genes predicted by FBA or MOMA should be excluded from the gene knockout sites list.

For evaluating a high value, different standards can be defined when necessary, e.g., 0.4 mmol•gDCW^-1^•h^-1^ is set in this study. Generally, more potential targets are obtained when a lower standard is adopted.


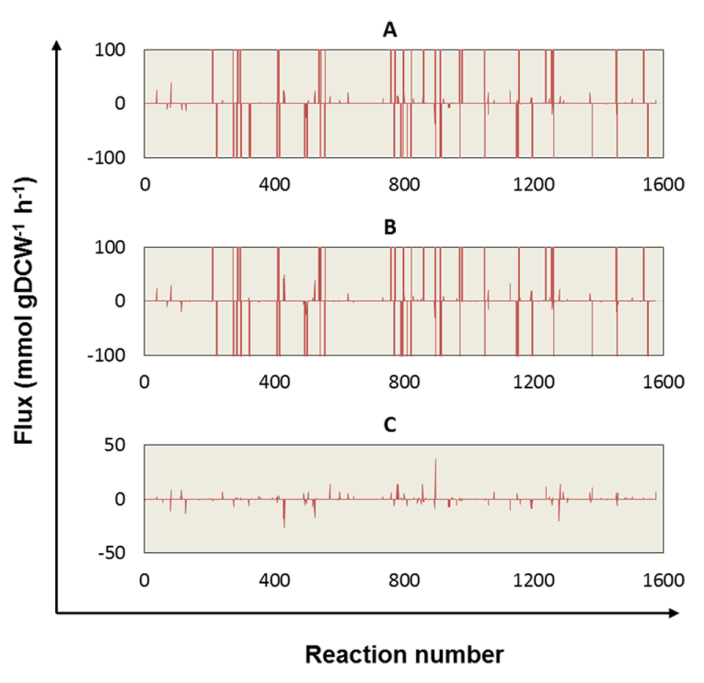


**Figure S1** **Flux distribution comparison analysis.** (A) The flux distribution of the metabolic network with maximum growth rate as the objective; (B) The flux distribution of the metabolic network with maximum IPP formation as the objective; (C) The difference of flux distribution of the metabolic network between A and B.


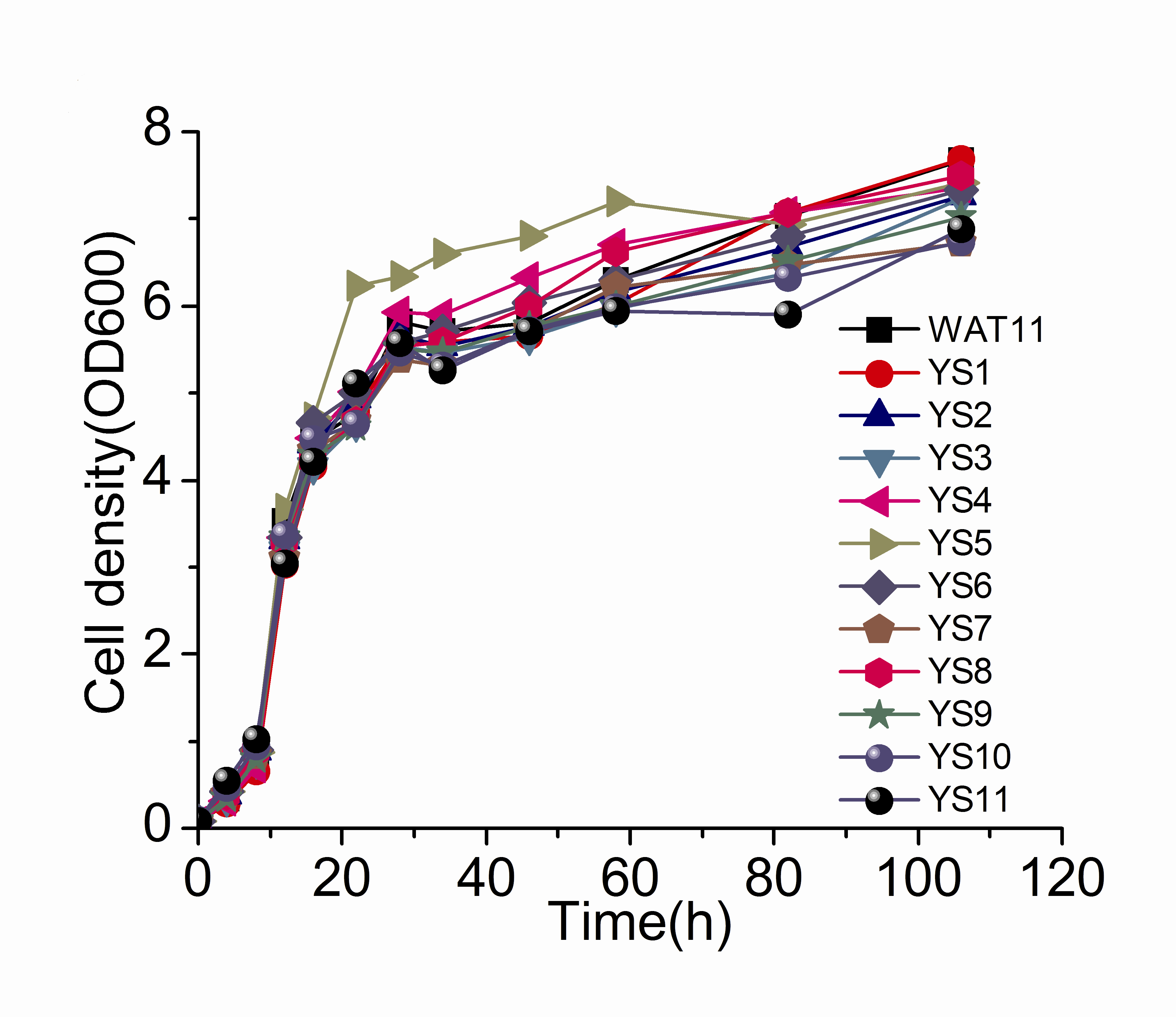


**Figure S2** **The growth property of the wild type WAT11 strain and single mutants**

**Table S1**  **Primers used in this study**

| **No.** | **Name** | **Sequence (5’ to 3’)** |
| --- | --- | --- |
| 1 | CRE-F | GAATTCATGTCCAATTT ACTGACC |
| 2 | CRE-R | GAGCTCCTAATCGCCATCTTCCAG |
| 3 | ADS-F | GGATCCATGTCACTTACAGAAG |
| 4 | ADS-R | CTCGAGTTATATACTCATAGG ATAAA |
| 5 | alt2-ORF-P1 | GTAAGAGGAGCTATTCCAACCAGAG |
| 6 | alt2-ORF-P2 | ATATTAAGGGTTGTCGACCTGCATATCTTCCGGTGTAGCGGGC |
| 7 | alt2-ORF-P3 | GATCTGCCGGTCTCCCTATAGTG CAGGACAAGCTGTGGTTGATTT |
| 8 | alt2-ORF- P4 | CCAGTCTTGAATCCATTTAGTCCCT |
| 9 | alt2-KanMX4-P2’ | GCCCGCTACACCGGAAGATAT GCAGGTCGACAACCCTTAATAT |
| 10 | alt2-KanMX4-P3’ | AAATCAACCACAGCTTGTCCTG CACTATAGGGAGACCGGCAGATC |
| 11 | ctp1-ORF- P1 | TTGCACTCGTTTCTGGCAGG |
| 12 | ctp1-ORF- P2 | ATATTAAGGGTTGTCGACCTGC TGATTGCTTCAAAAGGAGTCACTGC |
| 13 | ctp1-ORF- P3 | GATCTGCCGGTCTCCCTATAGTG GTCCGTGATAAAGGATTTTCTGGTC |
| 14 | ctp1-ORF- P4 | CACCTTTCCAAAACGTCTTTAACCC |
| 15 | ctp1-KanMX4- P2’ | GCAGTGACTCCTTTTGAAGCAATCAGCAGGTCGACAACCCTTAATAT |
| 16 | ctp1-KanMX4-P3’ | GACCAGAAAATCCTTTATCACGGACCACTATAGGGAGACCGGCAGATC |
| 17 | gre3-ORF- P1 | TAGTCGGCTTAGGGTGCTGGAAAAT |
| 18 | gre3-ORF- P2 | ATATTAAGGGTTGTCGACCTGC GGTGATGTGACCTTTCTTCTCGTCA |
| 19 | gre3-ORF- P3 | GATCTGCCGGTCTCCCTATAGTG GTAGTTGCTTACTCCTCCTTCGGTC |
| 20 | gre3-ORF- P4 | GAATTTACCATCCAACCAGGTCCAT |
| 21 | gre3-KanMX4-P2’ | TGACGAGAAGAAAGGTCACATCACCGCAGGTCGACAACCCTTAATAT |
| 22 | gre3-KanMX4-P3’ | GACCGAAGGAGGAGTAAGCAACTACCACTATAGGGAGACCGGCAGATC |
| 23 | hxk1-ORF- P1 | TTTAGGTCCAAAGAAACCACAGGCT |
| 24 | hxk1-ORF- P2 | ATATTAAGGGTTGTCGACCTGC CCATAAAGTCCTTCAAAGAGTCGGC |
| 25 | hxk1-ORF- P3 | GATCTGCCGGTCTCCCTATAGTG TCGAGGATGATCCATTTGAAAACTT |
| 26 | hxk1-ORF- P4 | TTTTTTCGGACAATGCAGCAATAAC |
| 27 | hxk1-KanMX4-P2’ | GCCGACTCTTTGAAGGACTTTATGGGCAGGTCGACAACCCTTAATAT |
| 28 | hxk1-KanMX4-P3’ | AAGTTTTCAAATGGATCATCCTCGACACTATAGGGAGACCGGCAGATC |
| 29 | hxk2-ORF- P1 | CCAAAAAAACCACAAGCCAGAAAGG |
| 30 | hxk2-ORF- P2 | ATATTAAGGGTTGTCGACCTGC CAAAGAGTCGGCAATAAATTCCCAC |
| 31 | hxk2-ORF- P3 | GATCTGCCGGTCTCCCTATAGTG CCCAGCCAGAATCGAGGAAG |
| 32 | hxk2-ORF- P4 | AATAACAGCGGCACCAGCAC |
| 33 | hxk2-KanMX4-P2’ | GTGGGAATTTATTGCCGACTCTTTGGCAGGTCGACAACCCTTAATAT |
| 34 | hxk2-KanMX4-P3’ | CTTCCTCGATTCTGGCTGGGCACTATAGGGAGACCGGCAGATC |
| 35 | idp1-ORF- P1 | ATCTCGTGACGCCACCTCCG |
| 36 | idp1-ORF- P2 | ATATTAAGGGTTGTCGACCTGC GGCCACACCACTGCCCTTGT |
| 37 | idp1-ORF- P3 | GATCTGCCGGTCTCCCTATAGTG GGATTTGGCTCCTTAGGTTTGATGA |
| 38 | idp1-ORF- P4 | CAACGGCATCCAAAAATTCTTCTGT |
| 39 | idp1-KanMX4-P2’ | ACAAGGGCAGTGGTGTGGCCGCAGGTCGACAACCCTTAATAT |
| 40 | idp1-KanMX4-P3’ | TCATCAAACCTAAGGAGCCAAATCCCACTATAGGGAGACCGGCAGATC |
| 41 | ser1-ORF- P1 | AGAGAGGAACCACAACATTTCGGAG |
| 42 | ser1-ORF- P2 | ATATTAAGGGTTGTCGACCTGC AAGATAACTTCAGCAGGAACGTGCA |
| 43 | ser1-ORF- P3 | GATCTGCCGGTCTCCCTATAGTG TGGGAGTACCAATCACCCCTATTGC |
| 44 | ser1-ORF- P4 | GGAGGCTCTGAACCCACCAACTGA |
| 45 | ser1- KanMX4-P2’ | TGCACGTTCCTGCTGAAGTTATCTTGCAGGTCGACAACCCTTAATAT |
| 46 | ser1-KanMX4-P3’ | GCAATAGGGGTGATTGGTACTCCCACACTATAGGGAGACCGGCAGATC |
| 47 | ser2-ORF- P1 | CCCAAAAGAAACCATCGACCAGA |
| 48 | ser2-ORF- P2 | ATATTAAGGGTTGTCGACCTGC TTCAACACCAGCATAAGCGGCA |
| 49 | ser2-ORF- P3 | GATCTGCCGGTCTCCCTATAGTG AACAAAAGCTAGAGGTCACCAAGGG |
| 50 | ser2-ORF- P4 | CGTTACCACCGTCACCCACCATA |
| 51 | ser2- KanMX4-P2’ | TGCCGCTTATGCTGGTGTTGAAGCAGGTCGACAACCCTTAATAT |
| 52 | ser2-KanMX4-P3’ | CCCTTGGTGACCTCTAGCTTTTGTTCACTATAGGGAGACCGGCAGATC |
| 53 | ser33-ORF- P1 | CTGGCTCTCCTGGTGCAGTCTCAAC |
| 54 | ser33-ORF- P2 | ATATTAAGGGTTGTCGACCTGC ACGGATCTTGAATTGGAGAATGGCG |
| 56 | ser33-ORF- P3 | GATCTGCCGGTCTCCCTATAGTG AAGCCGTCAAGGCCAACAAA |
| 57 | ser33-ORF- P4 | GCGATCTCGCCGTGAGAATC |
| 58 | ser33-KanMX4-P2’ | CGCCATTCTCCAATTCAAGATCCGTGCAGGTCGACAACCCTTAATAT |
| 59 | ser33-KanMX4-P3’ | TTTGTTGGCCTTGACGGCTTCACTATAGGGAGACCGGCAGATC |
| 60 | ser3-ORF- P1 | AATCTTTCATGAATACCGTTCCACAGC |
| 61 | ser3-ORF- P2 | ATATTAAGGGTTGTCGACCTGC GGAGAAAGGCGAGTTGAAAACAGCA |
| 62 | ser3-ORF- P3 | GATCTGCCGGTCTCCCTATAGTG ACATTCCATCTTTGATCCAAGCCGT |
| 63 | ser3-ORF- P4 | CGGTCTTCAAAACACCTGGTACATT |
| 64 | ser3-KanMX4-P2’ | TGCTGTTTTCAACTCGCCTTTCTCCGCAGGTCGACAACCCTTAATAT |
| 65 | ser3-KanMX4-P3’ | ACGGCTTGGATCAAAGATGGAATGTCACTATAGGGAGACCGGCAGATC |
| 66 | sor1-ORF- P1 | TCGAGCAAAGACCAATCCCTACCAT |
| 67 | sor1-ORF-P2 | ATATTAAGGGTTGTCGACCTGC CGACACAAGCGCCCTCTTCATAACT |
| 68 | sor1-ORF- P3 | GATCTGCCGGTCTCCCTATAGTG GCTACAGAGAGCAAAAGATTTCGGA |
| 69 | sor1-ORF-P4 | ACAGCGTCACGATAATCACCGAATG |
| 70 | sor1-KanMX4-P2’ | AGTTATGAAGAGGGCGCTTGTGTCGGCAGGTCGACAACCCTTAATAT |
| 71 | sor1-KanMX4-P3’ | TCCGAAATCTTTTGCTCTCTGTAGCCACTATAGGGAGACCGGCAGATC |
